# A Japanese family with popliteal pterygium syndrome

**DOI:** 10.3109/23320885.2015.1038347

**Published:** 2015-04-24

**Authors:** Motoki Katsube, Koh-ichiro Yoshiura, Kenji Kusumoto

**Affiliations:** 1 Department of Plastic and Reconstructive Surgery, Kansai Medical University, Hirakata, Japan; 2 Department of Human Genetics, Nagasaki University Graduate School of Biomedical Sciences, Nagasaki, Japan

**Keywords:** Cleft lip and palate, IRF6, popliteal pterygium syndrome, van der Woude syndrome

## Abstract

We investigated a family in which the mother and a daughter suffered from popliteal pterygium syndrome (PPS). Mutation in the interferon regulatory factor 6 (IRF6) gene was detected in the mother and daughter. This is the second report of a family case with mutation in the IRF6 gene in Japanese patients with PPS.

## Introduction

Popliteal pterygium syndrome (PPS) is an autosomal dominant disorder that was first reported in 1869 [1] and is characterized by multiple anomalies of the face, extremities, and genitalia. PPS is a rare syndrome with an incidence of ∼ 1 in 300,000 live births [2]. To our knowledge, only one family case with a gene mutation has been reported in Japan [3].

It is reported that mutations in the interferon regulatory factor 6 (IRF6) gene cause PPS [4]. It is recognized that PPS and van der Woude syndrome (VWS) are allelic, based on mutations in the IRF6 gene causing either PPS or VWS. We investigated a family in which the mother and a daughter suffered from PPS. Mutation screening in the IRF6 gene was performed in the mother and daughter. We report the results of the research and discuss PPS. This is the second report of a family case with mutation in the IRF6 gene in Japanese patients with PPS.

## Case report

**Figure 1.**
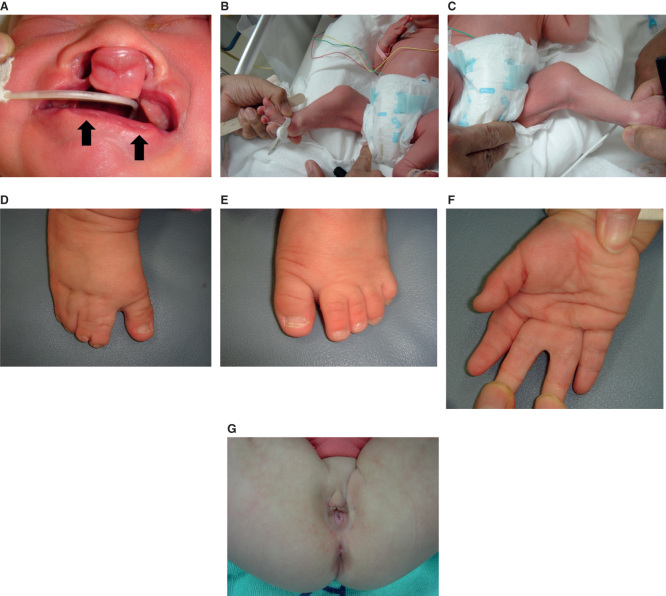
Illustrations showing the phenotypic appearance of the proband: (*a*) bilateral cleft lip and lower lip pits (arrows); (*b, c*) popliteal pterygium; (*d*) right foot complete cutaneous syndactyly between the second and third toes and the fourth and fifth toes and partial cutaneous syndactyly between the third and fourth toes; (*e*) left foot partial cutaneous syndactyly between the second and third toes; (*f*) hand cutaneous partial syndactyly; and (*g*) hypoplastic labia major.

**Figure 2.**
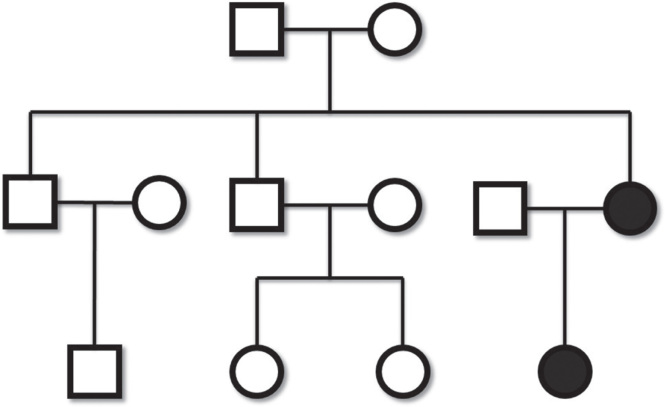
Pedigree of the family is shown. Filled symbols represent affected individuals.

Two individuals suffering from PPS appeared in two generations in a family. The proband, a 3-year-old girl, had bilateral cleft lip and palate (CLP), lower lip pits, right hand partial cutaneous syndactyly between the middle and ring fingers, right foot complete cutaneous syndactyly between the second and third toes and between the fourth and fifth toes and partial cutaneous syndactyly between the third and fourth toes, left foot partial cutaneous syndactyly between the second and third toes, bilateral equinovarus, bilateral popliteal pterygium, and hypoplastic labia major (Figure 1). We subsequently performed bilateral cheiloplasty by a modified de Haan’s method and removed the lower lip pits when the baby was 4 months old, and performed palatoplasty by a modified push back method when the baby was 1 year and 4 months old. Orthopedists performed bilateral popliteal pterygium repair by Z plasty and bilateral equinovarus repair when the baby was 11 months old. We performed repair of right hand syndactyly between the middle and ring fingers using dorsal flap, zigzag flaps, and skin graft from the right foot, and repair of right foot syndactyly between the second and third toes and the fourth and fifth toes using dorsal flap, zigzag flaps, and skin graft when the baby was 1 year and 10 months old. The mother of the proband has had corrected bilateral CLP, lower lip pits, and right foot syndactyly in infanthood. She did not have any other anomalies. Other members of the family were phenotypically normal (Figure 2).

**Figure 3.**
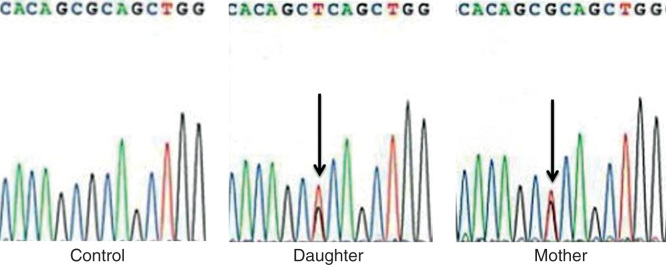
Sequence tracks from each individual are shown with an arrow pointing to the mutation. Left: control; center: daughter; and right: mother.

Blood samples were collected under informed consent from the proband and her mother. Mutation screening in the IRF6 gene was performed with an automated sequencer (Model 3130x, Applied Biosystems, USA). A missense mutation, c.250C>A, in IRF6 exon 4 was found in both of them (Figure 3).

## Discussion

VWS, which has clinical manifestations common to PPS, is an autosomal dominant disorder characterized by CLP and lip pits. VWS is the most common oral cleft syndrome and accounts for 2% of complete CLP cases. The genetic localization for VWS was reported to be mutations in chromosome 1q32-q41 [5] or 1q34 [6]. In 1999, it was reported that the genetic localization for PPS was mutations in chromosome 1q32, the same as in VWS, and they are allelic [7]. In 2002, it was reported that mutations in the IRF6 gene cause either PPS or VWS [8]. In 2014, it was reported that mutations in the GRHL3 gene cause VWS [9].

Mutations in IRF6 have been detected in some studies [8,10]. A total of 219 mutations in VWS and 36 mutations in PPS have been reported [8]. The distribution of mutations in IRF6, in VWS, and in PPS is non-random. Protein truncation mutations are common in VWS but are rare in PPS. The missense mutations are distributed between the DNA-binding domain and the protein-binding domain in VWS, while they are found in the DNA-binding domain in PPS [8]. Cases of either VWS or PPS caused by the same mutation in a family have been reported [11]. However, the relationship between clinical phenotypes and mutations in IRF6 has been unclear, as have the differences between mutations in IRF6 in VWS and in PPS. The mutation Arg84Cys is relatively common in PPS [4], but there are some reports that it is found either in VWS or in PPS [4,8]. It is thus difficult to distinguish between VWS and PPS only from the mutations in IRF6, so uniting them into a group of IRF6-related disorders is advocated [12].

There are some patients in which diagnosis of VWS or PPS is difficult. However, diagnostic criteria of VWS and PPS were advocated [12]. To make a diagnosis of VWS, at least one of the following findings must be present: 1. lip pits and CLP; 2. lip pits alone and a first-degree relative with CLP; and 3. CLP and a first-degree relative with lip pits. To establish a diagnosis of PPS, an individual must have one or more of the following in addition to features of VWS listed above: popliteal pterygia, syndactyly, abnormal external genitalia, ankyloblepharon, pyramidal skin on the hallux, and intraoral adhesions. We could easily make a diagnosis of the girl as having PPS because of some significant features of this condition. Her mother had syndactyly in addition to features of VWS, so we diagnosed her as having PPS.

The penetrance is high (92%) in VWS, while it is unclear in PPS. In view of the possibility of recurrence in the next generation, it is essential to recognize the presence of IRF6 mutations. Mutations in the IRF6 gene cause multiple phenotypes, so further investigations including mutation screening in the IRF6 gene are necessary.

**Acknowledgment** The authors thank Professor K Yoshiura, Nagasaki University, for his cooperation in the genetic screening.

***Declaration of interest:*** The authors report no conflicts of interest. The authors alone are responsible for the content and writing of the paper.
